# Evaluating Pictures of Nature and Soft Music on Anxiety and Well-Being During Elective Surgery

**DOI:** 10.2174/1874434601812010058

**Published:** 2018-04-24

**Authors:** Elinor Nielsen, Ingrid Wåhlin, Gunilla Hollman Frisman

**Affiliations:** 1 Department of Anesthesiology and Intensive Care, Vrinnevi Hospital Norrköping, Norrköping, Sweden; 2 Research Section, Kalmar County Council, S-391 85 Kalmar, Sweden and Linnaeus University, Faculty of Health and life Sciences, Växjö, Kalmar, Sweden; 3 Department of Anesthetics, Operations and Speciality Surgery Center and Medical and Health Sciences, Division of Nursing Sciences. Linköping University, 581 85 Linköping, Sweden

**Keywords:** Anesthesia, Awake, Distraction, Intervention, Music, Pictures

## Abstract

**Background:**

Patients going through surgery being awake often have a sense of anxiety and need support to relax.

**Objective:**

The aim of this study was to investigate whether looking at pictures of natural scenery could reduce anxiety and pain and increase relaxation and well-being being awake during the elective surgery.

**Methods:**

This three-arm, randomized intervention study consisted of one group viewing pictures of natural scenery, one group listening to soft instrumental music, and one control group without distraction, all adult patients (n=174). The State Trait Anxiety Inventory short form and a visual analogue scale on well-being were used as well as sedation treatment if necessary.

**Results:**

No differences related to anxiety after surgery were found among the three groups. When controlling for the effect of sedative treatment, however, patients without sedation had a lower degree of anxiety postoperatively (p=0.014). Younger patients had a higher degree of anxiety and lower degree of postoperative relaxation and well-being.

**Conclusion:**

Viewing pictures of natural scenery while being awake during elective surgery is as relaxing as listening to soft instrumental music. Offering nature scenery pictures for patients to view could be relaxing during the elective surgery.

## 1 INTRODUCTION

Patients undergoing surgery often have a sense of anxiety and stress [1, 2]. A higher level of preoperative anxiety has been associated with increased postoperative pain [3], prolonged recovery [4], increased hypotension after spinal anesthesia [5], and a greater risk of in-hospital mortality or major morbidity [6].

Spinal, epidural, or local anesthesia offers many advantages for the patient because it is less risky as compared to general anesthesia. However, these anesthesia options may be stressful and anxiety inducing because the patient is awake during surgery, so it is important to establish and maintain the patient’s comfort [7, 8]. The patient needs security during this exposed situation and can communicate with the nurse anesthetist or physician during the procedure [9]. Mediating a sense of comfort and keeping the patient focused are achieved by continuously giving the patient information, which is important for establishing the reassurance the patient needs [10]. In addition to reducing anxiety through establishing trust between the patient and the nurse anesthetist, different interventions have been tested for distracting patients to reduce the anxiety and stress [11-13] related to the surgery.

Nursing intervention to relieve unpleasant experiences has long relied on music. Music can also be used as a pleasant distraction to keep the patient from thinking about the situation they are in while undergoing surgery [14, 15]. The experience and effect of listening to music may be influenced by the choice of music as well as the patient’s earlier experience of it. Patient age, gender, and cultural background may also affect responses [16]. The music can be listened to using speakers or earphones, and it is preferred that the patient can change the volume at will [17].

Instrumental music with a rhythm of about 60–80 beats per minute has positive relaxing effects on anxiety, pain, and stress [18] and also positively influences circulation and respiration [15, 16]. Furthermore, relaxing music in a preoperative setting decreases anxiety preoperatively [19, 20] more than sedative oral premedication does [21]. In one study, patients listening to music while undergoing mastectomy had decreased mean arterial blood pressure, anxiety, and pain compared to patients in a control group [22]. Likewise, anxiety was reduced among patients listening to music during ambulatory surgery [23, 24]. Other distraction interventions used are audiovisual distractions. In one case study, Athanassoglou *et al*. [7] reported that patients undergoing limb microvascular orthoclastic surgery appreciated audiovisual distractions like watching TV or using the internet. They felt relaxed, which was also reported to be beneficial during the recovery.

Furthermore, seminal research on the sense of using colors in health care was performed by Ulrich *et al*. during the 1990s. Colors are used as distractors because they have different effects on people [25-27] including physiologically positive influences on lowering blood pressure and pulse rate. Green space, in general, has a positive health impact on stressful events in life [25, 28]. Viewing calm green pictures of natural scenery has been used in health care as a way to decrease stress and increase well-being [25, 26, 29] while challenging pictures have had the opposite effect [25, 26].

Patients undergoing bone marrow aspiration and biopsy who viewed pictures of nature scenery filled with green experienced less pain compared to patients who received standard care, although the difference was not statistically significant [29]. Natural scenery has also reduced anxiety about dental procedures and pain [30], and live plants and pictures of plants reduce the patient stress level in the waiting room [31]. The artificial natural scenery is a safe and low-cost distractor that has been used only to a limited extent. To our knowledge, there is no study using natural scenery as a distractor among adult awake patients undergoing elective surgery. The effect of music is however, better documented [4]. Thus, the objective of this study was to investigate whether looking at pictures of natural scenery could reduce anxiety and pain and increase relaxation and well-being being awake during elective surgery.

## 2 MATERIALS AND METHODS

This three-arm, randomized intervention study to evaluate the effect of looking at pictures of nature being awake during elective surgery was performed at three hospitals in Southern Sweden.

### 2.1 Participants

Inclusion criteria were adult patients over age 18 years planning to undergo urological, gynecological, or orthopedic surgery and who were fluent in the Swedish language. Patients with decreased vision or dementia were excluded.

Patients were included from three hospitals in Southeast of Sweden. Which patient group that should be included at each hospital were discussed and selected in collaboration with anesthesiologists, anesthesia nurses and surgeons at the respective hospital. These decisions were primarily based on which patient group at each hospital that most often was operated without general anesthetic. Urologic patients were selected at one hospital while orthopedic and surgical patients, respectively were selected at the other two hospitals. All patients who were operated from 2013-2015 and met the inclusion criteria, but not exclusion criteria were invited to participate in the study through an information letter when they arrived at the operation theater. High workload caused that some patients not received any request to participate but 225 patients were asked to participate in the study. The patient information consisted of the aim of the study, that the study was randomized into three groups, and what the three groups were (one group looking at pictures of natural scenery on an iPad, one listening to soft instrumental music during the surgery, and one control group without distraction). Furthermore, patients were asked to complete a questionnaire before and after the surgery. They were informed of the voluntary and confidential nature of the research and that they could withdraw their participation at any time. The patients thereafter were asked to provide a written informed consent.

### 2.2 Intervention

All patients who participated were randomized into the three groups. The randomization was performed through coded questionnaires stating the group the patient belonged to. These questionnaires were placed in coded envelopes. The patients who were randomized to look at pictures of natural scenery viewed the images on an iPad placed on an arm attached to the operating table so that the patient could see the pictures while lying on the operation table Fig. (**1**). Nature scenery pictures with calm green vegetation of the Swedish landscape without any animals were selected due to earlier study results reporting increased psychological well-being [25, 26]. There were 70 pictures and each picture was shown 45 seconds on the Ipad. Patients randomized to listen to music listened to soft instrumental music, MusiCure, music as medicine, with a rate of about 60-80 beats/minute, which was earlier reported to have positive effects through sensory stimulation on patients in health care. The music is composed from the evidence of patient experience and the CD number 5 (Seasons) from MusiCure collection composed by Niels Eje was used [32]. The patient listened to the music on a CD player using earphones and with the ability to control the volume themselves, meaning that the patient and the physician and nurses did not disturb each other. The patients who were randomized to the control group received care without distraction. There is a routine in Sweden that patients undergoing surgery without general anesthesia, recieve low doses of anesthesia (Propofol) or analgesia (*e.g.* Fentanyl) as supplement to local anesthesia, if feeling pain or discomfort. The need for this is usually assessed by the anesthetic nurse. All patients received medical drugs as described above when considered in need for this.

### 2.3 Description of Questionnaires

The questionnaire contained questions about patient demographic characteristics and was developed for this study. The following variables were included: age, surgery, if the patient had gone through surgery being awake earlier, anesthesia, and use of analgesics and/or sedation medication as documented by the anesthesia nurse caring for the patient. Where analgesics and/or sedation medications were used, the mean doses were analyzed to identify any differences between the intervention and the control groups.

The intervention group looking at pictures of natural scenery was asked about their wish to look at the pictures again if they underwent surgery while awake in the future. If the response was positive, they were asked if they wanted to bring their own pictures. The group listening to music was asked if they preferred to listen to music again and, if so, if they would prefer to bring their own music.

The questionnaire also consisted of the State Trait Anxiety Inventory (STAI) short form, measuring anxiety experienced by the patient before and after the surgery prior to discharge. The STAI short form consists of six possible states of anxiety: being calm, tense, upset, relaxed, content, or worried. The STAI short form is graded on a four-point scale, with a scale range from 6-24 points and a higher score meaning a higher degree of anxiety. The STAI short form is valid (the correlation between the full-length STAI and the short form is r = .95) and reliable (Cronbach’s alpha = .82) [33]. The STAI short form was used previously in similar studies allowing a comparison with the results of the current study.

Visual Analogue Scales (VAS) were used to measure anxiety, well-being, relaxation, and pain after the surgery. The VAS scale increments ranged from 1–100 mm. Patients were asked to mark an X to indicate how they felt during or after the surgery. Higher values indicate a greater extent of anxiety, well-being, pain, and level of relaxation, respectively [34]. The VAS for measuring pain has good reliability and validity [35], and VAS scales used in comparable studies have been shown to be suitable [18], providing the additional possibility for comparison with other research results.

### 2.4 Data Collection

No similar study exists, but for a power calculation, a possible difference between the groups was estimated to be two points on the STAI scale with a standard deviation of four. Power calculation then showed that 75 patients were needed in each of the three groups to achieve a power of 80% with p<0.05. A pilot study was performed on the eve of the data collection. All patients in the three groups were asked to complete the questionnaires described above. The STAI was completed before the surgery, and the STAI and the VAS scales were completed after surgery by nurses asking the patients to answer the scales, just before discharge in the recovery room.

### 2.5 Data Analysis

Descriptive statistics were used to analyze number, percentage, mean with Standard Deviation (SD), and median. Comparisons between the intervention groups and the control group according to age were made using one-way analysis of variance (ANOVA) test and for gender, anesthesia, surgical treatment and if having had surgery being awake earlier Kruskal Wallis test was used. To analyze the difference in medical treatment dosage during surgery MannWhitney U-test was used.

The Kruskal Wallis test was used to analyze differences in anxiety (STAI) preoperatively and anxiety (STAI), anxiety, well-being, relaxation and pain (VAS-scales) postoperatively. To control the effect of drug treatment with propofol and/or fentanyl linear regression analyzing with adjustment for propofol and fentanyl, respectively, was used, as these drugs may influence the experience of looking at pictures of nature scenery or listening to music. The mean age of 58 years was used as a cut-off to compare different age groups. The Mann-Whitney U-test was used to analyze the difference between anxiety, well-being, relaxation and pain (VAS-scales) among patients over and under med mean age 58 years and for gender difference according to preoperative anxiety (STAI). A p-value at p<0.05 was used for significance. Statistical Analysis software package (SPSS) version 22 was used.

### 2.6 Ethical Considerations

The study was approved by the Regional Ethical Committee and was conducted in accordance with the Declaration of Helsinki.

## 3 RESULT

Patients (n = 225) were asked to participate of whom n =174 (53% women) with a mean (SD) age of 58 (±17) years participated in the study. External drop out (n =51) was related to patients did not want to participate, was not interested to listen to music or wanted full anesthesia. More patients had spinal anesthesia compared to the other options (p<0.001), most had had orthopedic surgery (p<0.0001), and 58% (n=102) had previously undergone surgery while being awake (Table **1**). Of the participants, 70 were allocated to the intervention group looking at pictures of natural scenery, 56 to the intervention group listening to music, and 41 to the control group receiving routine care.

Sedative and analgesic medical drug treatment was administered to some patients. The control group received a significantly higher dose of propofol compared to the music group, a difference that disappeared when controlling for significance among the three groups (Table **2**).

No difference was found between the two intervention groups and the control group with respect to anxiety, well-being, relaxation, or pain after surgery (VAS scales; Table **3**), even when controlling for drug treatment with propofol and/or fentanyl. There was, however, a tendency to a difference regarding relaxation when adjusting for propofol (p=0.058), meaning a slightly higher degree of relaxation in the intervention group looking at pictures.

When controlling for the effects of propofol on the difference in anxiety before and after surgery as measured with STAI, patients who received propofol had a significantly higher degree of anxiety after surgery compared to those who did not (p=0.014). For treatment with fentanyl or propofol and fentanyl, no difference was found.

Patients under age 58 years had a higher degree of anxiety (p>0.001) and lower degree of relaxation and well-being (p=0.019 and p=0.003, respectively) compared to patients over 58 years. Women had a higher degree of preoperative anxiety than men (p=0.045).

Of the patients in the intervention group looking at pictures of natural scenery, 79% (n=68) said that they would look at pictures again; of those listening to music, 68% (n=54) preferred to listen to music if undergoing surgery again. For any upcoming surgery, 7% (n=6) preferred bringing their own pictures, and 11% (n=9) preferred bringing their own music.

## 4 DISCUSSION

Results showed no differences among the three groups regarding anxiety, well-being, relaxation, or pain after surgery. However, both the music intervention and the picture intervention groups received less sedation with propofol during the surgery compared with the control group. This difference may indicate that looking at pictures of natural scenery or listening to soft instrumental music can reduce anxiety during surgery, even if no significant differences could be measured postoperatively in the present study. However, patients who received propofol had a significantly higher degree of anxiety after surgery compared to those who did not, which may suggest that a distracting intervention is as calming as propofol treatment. In a previous study [36] aimed at determining the effectiveness of a photographic display on reducing preoperative anxiety, significant differences between the photographic group and the control group were obtained only for the respiratory rate. A music intervention was not evaluated separately in that study, but anxiety was found to decrease when pictures were combined with music.

A majority of patients in the current study (79% in the picture intervention group and 68% in the music intervention group) stated that they would like to have the same intervention if they had to undergo surgery again. This result could be interpreted as the interventions having had some positive effects, even if differences were not statistically significant using the measurements employed in the study. Experiences and emotions during surgery are individual experiences related to pre-understanding and previous experiences [37]. Music in the operation room has been found to hinder the nurses’ ability to hear the surgeon's speech [38], so the music is often delivered to the patient by earphones. However, some patients would like to know what is going on during the surgery [37]. For those patients, looking at pictures could offer some distraction while still maintaining the opportunity to get information and participate in what is going on in the operation room. A recent interview study revealed that nurse anesthetists assumed that all patients were anxious prior to surgery [39]. Increased work experiences made it easier to handle anxious patients, and more experienced nurses valued nursing interventions and calming conversations prior to administering pharmaceuticals. This possibility has not, to our knowledge, been explored, although such comments were heard during the present study. Insufficient time related to high workloads has also been experienced to affect the first encounter with the patient and how intraoperative anxiety is addressed [39]. If the workload is high, it may be seen as faster and easier to offer sedation than another relaxing alternative, such as looking at pictures or listening to music. One positive effect that occurs when patients are distracted during the surgery may be less patient movement when lying on the operation table, which may be beneficial for the surgeon. This possibility has not, to our knowledge, been explored, although such comments were heard during the present study.

Women had more preoperative anxiety than men, and younger patients showed a higher degree of anxiety compared to older patients, what is in line with earlier reported results [40-42].

However, there are several limitations with this study. One limitation is that a decision was taken to terminate the study before reaching the estimated number of patients. This decision was related to a pronounced high workload at the operating theathers and a problem to perform any task beyond routine care. Despite this, the study involved a relatively large number of patients in comparison with other, similar studies and the result could hopefully be useful for designing future studies in the area.

Lack of difference between groups in the current study may be due to the fact that the effect was not captured by the parameters measured. Further research in the area may therefore, need to include parameters measuring stress during the actual procedure together with self-reported assessments afterward.

Another limitation in this study is related to the randomization procedure that did not work fully, probably because nurses found it difficult to first ask patients about interest in participating in a study about listening to music or watching pictures of nature during the operation and then go back to the patient and tell them that they had been randomized to a control group with either music or pictures. The staff would have needed more information about the importance of following the randomization.

Another limitation was that the included patients were operated in various operation rooms where the iPad with pictures often had to be installed and readjusted for each patient. Because the study was conducted within the context of daily activities involving a large number of staff members, several staff members never had time to become skilled at installing the equipment in a fast and appropriate manner. This situation would probably have been different if the intervention had been managed by fewer staff members or by staff members who were familiar with the equipment. Furthermore, the absence of standardization for sedative medication may be seen as a limit. In Sweden, patient needs always must regulate drug administration, so even if the current purpose was to study patients without sedative medication, some of them asked for or needed it.

Physiological parameters such as signs of pain or anxiety not measured may also be seen as a limit. Moreover, in the future, it would be interesting to evaluate a combination of a picture and music intervention.

## CONCLUSION

Looking at pictures of natural scenery being awake during surgery is as relaxing as listening to soft instrumental music. This knowledge could be especially valuable for patients with poor hearing if they need distraction during surgery. Being aware that women, and perhaps also younger patients, have a tendency to heightened anxiety before surgery could also be valuable. Pictures of natural scenery should be studied also in other contexts because looking at natural scenery may have generalizable advantages.

## Figures and Tables

**Fig. (1):**
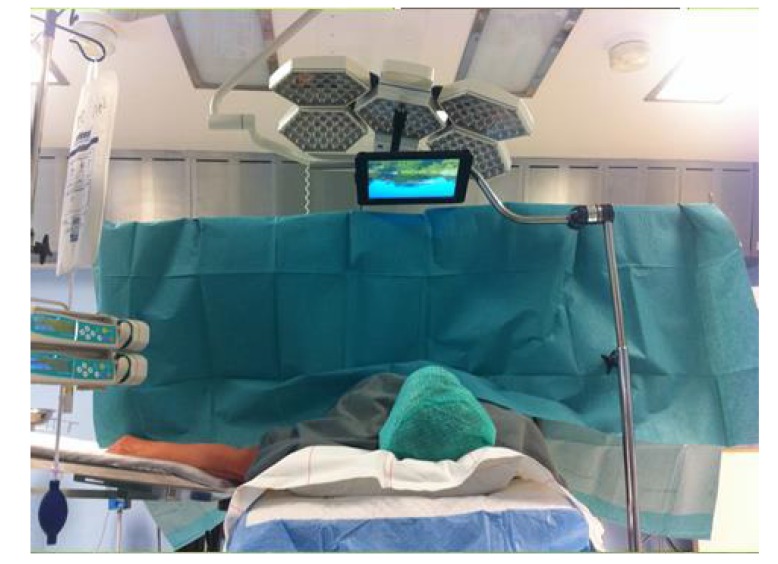
A patient in the intervention group looking at pictures of natural scenery on an iPad.

**Table 1:** Characteristics of patients in the intervention and control groups who underwent surgery while awake.

–	–	** Intervention Groups **			
–	** All Patients **	** Listening to Music **	** Looking at Pictures **	** Control Group **	** P value **
**Men/women, n (%)**	82 (53)/ 92 (47)	28 (48)/ 30 (52)	36 (50)/ 36 (50)	18 (41)/ 26 (59)	0.623
**Age, mean (SD)**	58 (17)	59 (17)	72 (16)	56 (18)	0.489
**Anesthesia**					0.004
**Spinal, n (%)**	80 (48)	28 (48)	41 (57)	11 (25)	
**Epidural, n (%)**	1 (0.6)	0	1 (1)	0	
**Local, n (%)**	86 (49)	28 (48)	28 (39)	30 (68)	
**Surgical treatment**					<0.0001
**Surgery, n (%)**	51 (29)	30 (52)	14 (19)	7 (16)	
**Urology, n (%)**	34 (19)	15 (26)	16 (22)	3 (7)	
**Gynecology, n (%)**	2 (1)	2 (3)	0	0	
**Orthopedics, n (%)**	87 (50)	11 (19)	42 (58)	34 (77)	
**Had surgery being awake earlier, n (%)**	102 (58)	37 (64)	40 (56)	25 (57)	0.534

SD, Standard Deviation. Missing values for age, n=1, and for anesthesia, n=7.

**Table 2:** Differences in medical treatment during surgery in the intervention groups versus the control group.

** Medical ** ** Treatment **	** Looking at Pictures **	** Listening to Music **	** Control Group **	** P value ** ** looking at pictures *vs*. control group **	** P value listening to music *vs*. control group **	** P value looking at pictures *vs*. listenting to music **
Propofol mg, m (SD)	88 (77)	90 (84)	137 (101)	0.056	0.042	0.230
Fentanyl, µg, m (SD)	71(27)	69 (27)	63 (33)	0.121	0.258	0.230

In total 82 patients got propofol and 60 patients got fentanyl.

m=mean, SD=Standard Deviation. Difference was calculated with MannWhithey U-test.

**Table 3:** Anxiety, relaxation, well-being and pain before and after surgery in patients looking at pictures, listenting to music or being in the control group.

	** Looking at pictures of the nature ** ** n=70 ** ** m (SD) **	** Listening to Music ** ** n=56 ** ** m (SD) **	** Control Group ** ** n=41 ** ** m (SD) **	** P value **
**Anxiety STAI** **Before surgery**	13.9 (2.0)	14.0 (1.3)	13.6 (1.7)	0.100
**After surgery**	14.5 (0.9)	14.9 (3.7)	14.4 (1.3)	0.563
**Anxiety, VAS after surgery**	15.1 (22.1)	16.5 (22.3)	15.0 (19.1)	0.802
**Relaxation, VAS after surgery**	82.9 (21.5)	80.5 (23.3)	81.9 (25.9)	0.557
**Well-being, VAS after surgery**	83.7 (19.2)	82.0 (20.3)	84.3 (16.8)	0.863
**Pain, VAS after surgery**	7.0 (14.0)	7.6 (13.6)	8.8 (14.3)	0.670

STAI, State Trait Anxiety Inventory; VAS, Visual Analog Scale. Differences are calculated with Kruskal Wallis test.

## References

[1] Wetsch W.A., Pircher I., Lederer W., Kinzl J.F., Traweger C., Heinz-Erian P., Benzer A. (2009). Preoperative stress and anxiety in day-care patients and inpatients undergoing fast-track surgery.. Br. J. Anaesth..

[2] Yilmaz M., Sezer H., Gürler H., Bekar M. (2012). Predictors of preoperative anxiety in surgical inpatients.. J. Clin. Nurs..

[3] Ocalan R., Akin C., Disli Z.K., Kilinc T., Ozlugedik S. (2015). Preoperative anxiety and postoperative pain in patients undergoing septoplasty.. B-ENT.

[4] Bradt J., Dileo C., Potvin N. (2013). Music for stress and anxiety reduction in coronary heart disease patients.. Cochrane Database Syst. Rev..

[5] Orbach-Zinger S., Ginosar Y., Elliston J., Fadon C., Abu-Lil M., Raz A., Goshen-Gottstein Y., Eidelman L.A. (2012). Influence of preoperative anxiety on hypotension after spinal anaesthesia in women undergoing Caesarean delivery.. Br. J. Anaesth..

[6] Williams JB, Alexander KP, Morin JF, Langlois Y, Noiseux N, Perrault LP

[7] Athanassoglou V., Wallis A., Galitzine S. (2015). Audiovisual distraction as a useful adjunct to epidural anesthesia and sedation for prolonged lower limb microvascular orthoplastic surgery.. J. Clin. Anesth..

[8] Hu P., Harmon D., Frizelle H. (2007). Patient comfort during regional anesthesia.. J. Clin. Anesth..

[9] Karlsson A.C., Ekebergh M., Mauléon A.L., Almerud Österberg S. (2012). “Is that my leg?” patients’ experiences of being awake during regional anesthesia and surgery.. J. Perianesth. Nurs..

[10] Berg K., Kaspersen R., Unby C., Hollman Frisman G. (2013). The interaction between the patient and nurse anesthetist immediately before elective coronary artery bypass surgery.. J. Perianesth. Nurs..

[11] Guo P., East L., Arthur A. (2012). A preoperative education intervention to reduce anxiety and improve recovery among Chinese cardiac patients: A randomized controlled trial.. Int. J. Nurs. Stud..

[12] Johnson B., Raymond S., Goss J. (2012). Perioperative music or headsets to decrease anxiety.. J. Perianesth. Nurs..

[13] Rosenfeldt F, Braun L, Spitzer O, Bradley S, Shepherd J, Bailey M

[14] Gooding L., Swezey S., Zwischenberger J.B. (2012). Using music interventions in perioperative care.. South. Med. J..

[15] Nilsson U. (2008). The anxiety- and pain-reducing effects of music interventions: A systematic review.. AORN J..

[16] Engwall M., Duppils G.S. (2009). Music as a nursing intervention for postoperative pain: A systematic review.. J. Perianesth. Nurs..

[17] Harikumar R., Raj M., Paul A., Harish K., Kumar S.K., Sandesh K., Asharaf S., Thomas V. (2006). Listening to music decreases need for sedative medication during colonoscopy: A randomized, controlled trial.. Indian J. Gastroenterol..

[18] Björkman I., Karlsson F., Lundberg A., Frisman G.H. (2013). Gender differences when using sedative music during colonoscopy.. Gastroenterol. Nurs..

[19] Kipnis G., Tabak N., Koton S. (2016). Background music playback in the preoperative setting: Does it reduce the level of preoperative anxiety among candidates for elective surgery?. J. Perianesth. Nurs..

[20] McClurkin S.L., Smith C.D. (2016). The duration of self-selected music needed to reduce preoperative anxiety.. J. Perianesth. Nurs..

[21] Bringman H., Giesecke K., Thörne A., Bringman S. (2009). Relaxing music as pre-medication before surgery: A randomised controlled trial.. Acta Anaesthesiol. Scand..

[22] Binns-Turner P.G., Wilson L.L., Pryor E.R., Boyd G.L., Prickett C.A. (2011). Perioperative music and its effects on anxiety, hemodynamics, and pain in women undergoing mastectomy.. AANA J..

[23] Ilkkaya N.K., Ustun F.E., Sener E.B., Kaya C., Ustun Y.B., Koksal E., Kocamanoglu I.S., Ozkan F. (2014). The effects of music, white noise, and ambient noise on sedation and anxiety in patients under spinal anesthesia during surgery.. J. Perianesth. Nurs..

[24] Palmer JB, Lane D, Mayo D, Schluchter M, Leeming R

[25] RS (1991). U. Effects of health facility interior design on wellness: Theory and scientific research.. J Health Care Design..

[26] Marcus C.C.B.M. (1999). U. Effects of gardens on health outcomes: Theory and research.. Healing gardens: Therapeutic benefits and design recommendations..

[27] Ulrich R.S.P.R. Influences of passive experiences with plants on individual well-being and health..

[28] van den Berg A.E., Maas J., Verheij R.A., Groenewegen P.P. (2010). Green space as a buffer between stressful life events and health.. Soc. Sci. Med..

[29] Lechtzin N, Busse AM, Smith MT, Grossman S, Nesbit S, Diette GB

[30] Tanja-Dijkstra K, Pahl S, White MP, Andrade J, May J, Stone RJ

[31] Beukeboom CJ, Langeveld D, Tanja-Dijkstra K

[32] Eje N. (2016). Musicure Copenhagenhttp://musicure.com/.

[33] Marteau T.M., Bekker H. (1992). The development of a six-item short-form of the state scale of the Spielberger State-Trait Anxiety Inventory (STAI).. Br. J. Clin. Psychol..

[34] Huskisson E.C. (1974). Measurement of pain.. Lancet.

[35] Lundeberg T., Lund I., Dahlin L., Borg E., Gustafsson C., Sandin L., Rosén A., Kowalski J., Eriksson S.V. (2001). Reliability and responsiveness of three different pain assessments.. J. Rehabil. Med..

[36] Gómez-Urquiza J.L., Hueso-Montoro C., Urquiza-Olmo J., Ibarrondo-Crespo R., González-Jiménez E., Schmidt-Riovalle J. (2016). A randomized controlled trial of the effect of a photographic display with and without music on pre-operative anxiety.. J. Adv. Nurs..

[37] Engström Å, Boström J., Karlsson A.

[38] Weldon S.M., Korkiakangas T., Bezemer J., Kneebone R. (2015). Music and communication in the operating theatre.. J. Adv. Nurs..

[39] Bengtsson Y., Johansson A., Englund E. (2016). Nurse anaesthetists’ experiences of the first intraoperative meeting with anxious adult patients: An interview study.. Nord. J. Nurs. Res..

[40] Mitchell M. (2012). Anxiety management in minimal stay surgery.. Nurs. Times.

[41] Mavridou P., Dimitriou V., Manataki A., Arnaoutoglou E., Papadopoulos G. (2013). Patient’s anxiety and fear of anesthesia: Effect of gender, age, education, and previous experience of anesthesia. A survey of 400 patients.. J. Anesth..

[42] Nigussie S, Belachew T, Wolancho W

